# The effect of health insurance reform on the number of cataract surgeries in Chongqing, China

**DOI:** 10.1186/1472-6963-11-67

**Published:** 2011-03-26

**Authors:** Xiaofan Chen, Chunlin Chen, Yao Zhang, Rongdi Yuan, Jian YE

**Affiliations:** 1 Department of Ophthalmology, Institute of Surgery Research, Daping Hospital, Third Military Medical University, Chongqing, 400042, China; 2 Department of Epidemiology, College of Preventive Medicine, Third Military Medical University, Chongqing, 400038, China

## Abstract

**Background:**

Cataracts are the leading cause of blindness in China, and poverty is a major barrier to having cataract surgery. In 2003, the Chinese government began a series of new national health insurance reforms, including the New Cooperative Medical Scheme (NCMS) and the Urban Resident Basic Health Insurance scheme (URBMI). These two programs, combined with the previously existing Urban Employee Basic Health Insurance (UEBMI) program, aimed to make it easier for individuals to receive medical treatment. This study reports cataract surgery numbers in rural and urban populations and the proportion of these who had health insurance in Chongqing, China from 2003 to 2008.

**Methods:**

The medical records of a consecutive case series, including 14,700 eyes of 13,262 patients who underwent age-related cataract surgery in eight hospitals in Chongqing from January 1, 2003, to December 31, 2008, were analysed retrospectively via multi-stage cluster sampling.

**Results:**

In the past six years, the total number of cataract surgeries had increased each year as had the number of patients with insurance. Both the number of surgeries and the number of insured patients were much higher in the urban group than in the rural group. The rate of increase in the rural group however was much higher than in the urban group, especially in 2007 and 2008. The odds ratios of having health insurance for urban vs. rural individuals were relatively stable from 2003 to 2006, but it decreased in 2007 and was significantly lower in 2008.

**Conclusions:**

Health insurance appears to be an important factor associated with increased cataract surgery in Chongqing, China. With the implementation of health insurance, the number of Chongqing's cataract surgeries was increased year by year.

## Background

In China, cataracts are the leading cause of blindness [1-3], and there are currently approximately 2.5 million people with cataracts in China [4]. The only treatment is surgical removal. In recent years, with the promotion of "Vision 2020--the Right to Sight", under the guidance of and with the support of the Chinese Ministry of Health, Disabled Persons' Federation and non-governmental organisations at home and abroad, significant efforts have been made to promote the treatment of cataract blindness. For example, partial subsidies have been provided by the government and free surgeries have been donated by charitable societies. However, not everyone who needs surgical cataract treatment can benefit from subsidies or charity surgery. Therefore, additional measures to improve this situation are urgently needed.

In 2003, the Chinese government introduced a series of new national health insurance reforms, including the New Cooperative Medical Scheme (NCMS) and the Urban Resident Basic Health insurance scheme (URBMI). These two programs, combined with the previously existing Urban Employee Basic Health Insurance (UEBMI) program, aimed to make it easier for individuals to receive medical treatment.

We wanted to know whether the health insurance reforms have benefited cataract surgery. In this study, we retrospectively investigated the cataract surgery data for Chongqing from 2003 to 2008 to determine whether the health insurance reforms have had some effect on the number of cataract surgeries.

## Methods

### Study sample

This retrospective study used multi-stage cluster sampling. There are 31 districts in Chongqing. We selected 6 districts randomly from the 31 districts: the Yuzhong, Shapingba, Jiangbei, Jiulongpo, Dadukou, and Nanan districts. The second step was to select government-owned hospitals that had the ability to carry out cataract surgery in the above selected districts. We selected 4 tertiary hospitals and 4 secondary hospitals for this study. The tertiary hospitals were Daping Hospital of the Third Military Medical University in the Yuzhong district, the Second Affiliated Hospital of Chongqing Medical University in the Yuzhong district, Chongqing Third People's Hospital in the Yuzhong district, and Wujing Chongqing Zongdui Hospital in the Nanan district. The secondary hospitals were Jiangbei First People's Hospital of Chongqing in the Jiangbei district, Shapingba First People's Hospital of Chongqing in the Shapingba district, Chongqing Iron and Steel Company General Hospital in the Dadukou district, and the Staff-worker Hospital of the Chongqing Machine Tool Factory in the Jiulongpo district. The study was approved by the Institutional Review Boards at the participating institutions.

Consecutive case study analysis was applied to collect clinical data from the medical records of patients with age-related cataracts. The cases included 14,700 eyes of 13,262 patients who underwent surgical treatment at one of the above-mentioned hospitals between January 1, 2003 and December 31, 2008. The total number of patients, the geographic distribution and the health insurance status were retrospectively analysed for all cases.

### Categories of Cases

Based on medical history information, all patients were designated as urban residents or rural residents according to the designated geographic division. Based on the health insurance status of patients, such as UEBMI and URBMI for urban dwellers and NCMS for rural residents, the cases were divided into two groups: the insured group, which included patients with health insurance, and the uninsured group, which included patients without health insurance.

### Statistical Analysis

The data collection forms were developed by a team that included ophthalmologists and epidemiologists. All clinical data for the eyes of the age-related cataract patients included in this study were computerised and then rechecked by different staff members. Then, all data were analysed by chi-square tests with SPSS for Windows, version 13.0 (SPSS Inc, Chicago, IL, USA), and p < 0.05 was considered statistically significant.

## Results

**Figure 1:**
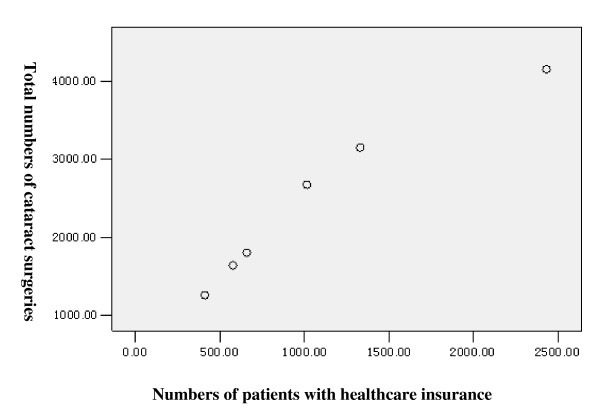
**Scatterplots of the number of cataract surgery Versus patients with health insurance**.

**Figure 2:**
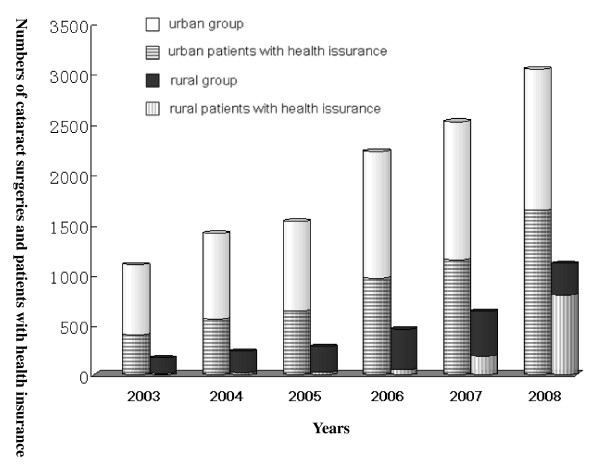
**Comparison of cataract surgery numbers and patients with health issuance in two groups**.

**Figure 3:**
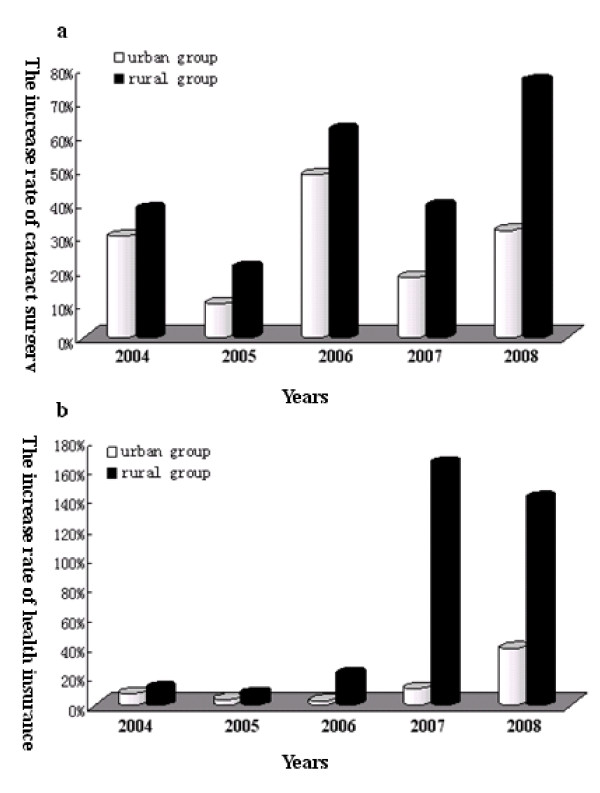
**Rates of increase of the numbers of surgeries and insured patients in two groups**.

In the past six years, the total number of cataract cases had increased each year as had the number of patients with health insurance (Table 1). These two values had a significant linear positive correlation (r = 0.971, p = 0.001) (Figure 1). Though the number of both cataract surgeries and insured patients in the urban group were much higher than in the rural group (Figure 2), the rate of increase in the rural group was much higher than in the urban group, especially in 2007 and 2008 (Figure 3). The odds ratios of having health insurance for urban vs. rural patients were 6.83 (95% CI, 3.83-12.18) in 2003, 6.93 (95% CI, 4.33-11.09) in 2004, 6.93 (95% CI, 4.57-10.51) in 2005, 6.04 (95% CI, 4.46-8.17) in 2006, 1.96 (95% CI, 1.63-2.37) in 2007 and 0.47 (95% CI, 0.40-0.54) in 2008 (Table 1).

**Table 1:** Medical insurance of cataract patients between urban and rural patients

year	Cataract surgery output			The eyes of urban patients			The eyes of rural patients			*OR (95% CI)*	*P *Value
			
	Total eyes	medical insurance	(%)	Total eyes	medical insurance	(%)	Total eyes	medical insurance	(%)		
2003	1264	410	32.44	1095	397	(36.26)	169	13	(7.69)	6.83(3.83-12.18)	<0.001
2004	1644	576	35.04	1411	556	(39.40)	233	20	(8.58)	6.93(4.33-11.09)	<0.001
2005	1807	658	36.41	1526	632	(41.42)	281	26	(9.25)	6.93(4.57-10.51)	<0.001
2006	2678	1014	37.86	2224	963	>(43.30)	454	51	(11.23)	6.04(4.46-8.17)	<0.001
2007	3153	1330	42.18	2523	1143	(45.30)	630	187	(29.68)	1.96(1.63-2.37)	<0.001

2008	4154	2432	58.55	3045	1640	(53.86)	1109	792	(71.42)	0.47(0.40-0.54)	<0.001

OR = odds ratios; CI = confidence interval

## Discussion

Cataracts are by far the most common cause of major vision loss, accounting for approximately half of all global cases of blindness. According to the World Health Organization (WHO), China is estimated to have the largest number of cataract-blind people in the world [5]. During the past three decades, China has experienced explosive economic growth and development, however, China's cataract surgical rate (CSR), which represents the number of cataract operations performed annually per one million of population, is among the lowest in the world and thus lags behind China's astonishing achievement in other areas.

Why is China's CSR so low with such remarkable economic progress? Among the many contributing factors, the major reason for the low CSR is the low availability of basic social services, particularly health insurance services, in rural areas. It is estimated that out of China's 1.3 billion citizens, more than 60% of China's population lives in rural regions [6], but more than 80% of the country's health resources are concentrated in large cities. This disparity is responsible for the immense gap in health insurance availability between rural and urban populations.

Prior to 2003, there was only one primary insurance program for the urban employed: the Urban Employee Basic Medical Insurance (UEBMI) program. In 2003, the Chinese government launched the New Cooperative Medical Scheme (NCMS), aimed to provide health coverage for the nation's entire rural population by 2010. The NCMS has witnessed a rapid expansion in coverage since its inception, beginning in only 310 out of China's 2861 rural counties in 2004 but expanding to 2451 counties by the end of 2007, accounting for 86% of all rural counties in China [7,8]. However, another population cohort, the 420 million urban residents without formal employment, was completely left out of the state health insurance safety net. Thus, in the second half of 2007, the Chinese government begun to implement the Urban Resident Basic Health Insurance scheme (URBMI), and the government wants to cover all urban residents by 2010. These three health insurance systems benefit all Chinese citizens [9-15].

Our study revealed that the total number of cataract surgeries had increased each year as had the number of patients with health insurance, suggesting that the availability of health insurance had a significant effect on the number of cataract surgeries. The rates of increase of the numbers of surgeries and insured patients in the rural group were much higher than in the urban group, especially in 2007 and 2008. The odds ratios of having health insurance between the urban and rural groups were relatively stable from 2003 to 2006, but it decreased in 2007 and was significantly lower in 2008. This difference may be related to the implementation of the health insurance reforms, as the NCMS was started in 2003 and expanded quickly up to 86% coverage by 2007, but the URBMI was piloted just in the second half of 2008. These changes are in line with Chinese governmental health insurance reform, indicating that health insurance may have some influence on the amount of cataract surgery in Chongqing.

This study has several limitations. First, not only health insurance reform could affect the number of cataract surgeries, many other factors, such as improved surgical outcomes, increased individual disposable income, enhanced life quality pursuiting, etc, could also have some effects on cataract surgery, but in this study, we could not exclude the other factors' role in the promotion of cataract surgery and make the causal association between insurance coverage and cataract surgery. Second, being a hospital-based retrospective study, we could not elucidate the coverage of health insurance among Chongqing people, therefore, we only investigated the correlation between the number of cataract surgeries and the number of patients with health insurance. These limitations should be addressed in future studies using surveys designed to allow us to further tackle the questions concerning health insurance and the outcomes among patients with cataracts in China.

## Conclusions

Health insurance appears to be an important factor associated with increased cataract surgery in Chongqing, China. With the implementation of health insurance, the number of Chongqing's cataract surgeries was increased year by year.

## Competing interests

The authors declare that they have no competing interests.

## Authors' contributions

XFC, CLC and YJ designed and supervised the study and provided statistical advice throughout the study. XFC analysed and interpreted the data. CLC drafted and finalised the manuscript. YZ and RDY revised the manuscript. All authors read and approved the final version of the manuscript.

## Pre-publication history

The pre-publication history for this paper can be accessed here:

http://www.biomedcentral.com/1472-6963/11/67/prepub
